# Out-of-reach rewards elicit human-oriented referential communicative behaviours in family dogs but not in family pigs

**DOI:** 10.1038/s41598-022-26503-5

**Published:** 2023-01-23

**Authors:** Paula Pérez Fraga, Boglárka Morvai, Linda Gerencsér, Fanni Lehoczki, Attila Andics

**Affiliations:** 1 Department of Ethology, Eötvös Loránd University (ELTE), Pázmány P. S. 1/C, Budapest, 1117 Hungary; 2 MTA-ELTE ‘Lendület’ Neuroethology of Communication Research Group, Hungarian Academy of Sciences-Eötvös Loránd University, Budapest, Hungary; 3 Doctoral School of Biology, Institute of Biology, Eötvös Loránd University, Budapest, Hungary

**Keywords:** Animal behaviour, Social behaviour, Psychology

## Abstract

Human-oriented referential communication has been evidenced not only in domestic but also in some wild species, however, the importance of domestication-unrelated species’ characteristics in the emergence of this capacity remains largely unexplored. One shared property of all species reported to exhibit referential communication is the efficient use of visual social signals. To assess the potential role of species-specific characteristics in the emergence of human-oriented referential communication, we compared similarly socialised companion animals from two domestic species: dogs, which rely heavily on conspecific visual social signals; and pigs, which do not. We used an out-of-reach reward paradigm with three conditions: both human and reward present, only human present, only reward present. Both species exhibited certain behaviours (e.g. orientation towards the human, orientation alternation between the human and the reward) more often in the human’s presence. However, only dogs exhibited those behaviours more often in the simultaneous presence of the human and the reward. These results suggest similar readiness in dogs and pigs to attend to humans but also that pigs, unlike dogs, do not initiate referential communication with humans. The ability to referentially communicate with humans may not emerge in mammals, even if domesticated companion animals, that lack certain species characteristics, such as efficient intraspecific visual communication.

## Introduction

Referential communication, the act of directing others' attention to specific entities in the environment is key for coordinating action with, asking for help from, or teaching about the world to social partners^1,2^_._ It is thought to have evolved in species with complex social systems and to bring clear benefits for both the sender and the receiver^3^. Human language constitutes a full-fledged referential communicative system^4^ and referentiality is also central in preverbal infants’ gestural communication (e.g. pointing)^5^. The functionally referential communicative capacities of non-human animals have attracted significant research attention^6,7^ with a special focus on the extent to which this type of communication is possible between animals and humans.

During the last decade, human-oriented functionally referential communication has been observed across a variety of mammals^8–13^. Domestication, even though certainly a booster, may not be a necessary prerequisite for this ability to emerge: some human-socialized wild animals have also been reported to perform human-oriented referential communicative behaviours (e.g. pointing, gaze alternation between the human and a desired target, etc.)^14–16^. But is domestication or intense human socialization a sufficient prerequisite? In support, almost all domestic species tested so far exhibit referential communicative behaviours towards humans^9–11,17,18^. Remarkably, however, all species for which referential communication with humans has been reported (dogs^17,19^, goats^9,20^ horses^10,21^, cats^18^, dolphins^12^, wolves^22^, kangaroos^15^, non-human primates^8,13^) use visual social signals efficiently and rely on them heavily for within-species communication and cooperation. This raises the alternative hypothesis that readiness for human-oriented referential communication may not emerge in species that lack certain species-specific characteristics such as the efficiency of intraspecific visual communication.

Dogs (*Canis familiaris*) and pigs (*Sus scrofa domesticus*) are domestic species with a comparably complex social system^23,24^ that, when kept as companion animals, are raised very similarly^25^. Their domestication histories, although certainly different in purpose (i.e. dogs were selected for cooperative tasks and pigs as livestock), have several points in common^26,27^, for example a close contact with humans^28,29^, as presumably the ancestors of both dogs and pigs scavenged leftovers around human settlements. Consequently, humans might have become important social stimuli for both species. Despite these similarities, pigs and dogs clearly differ in their use of visual social cues, among others. Dogs rely heavily on visual communication for intraspecific interactions^30,31^ and read human visual social cues efficiently without training ^32,33^. Pigs, on the contrary, do not often use visual signals for intraspecific communication^34–36^, perhaps partly because of their poor visual acuity (poorer than that of dogs and humans)^37^, or due to anatomical restraints such as the rigidity of their neck^38^. Pigs also fail to spontaneously follow human visual cues^39,40^ and seem less responsive to human facial patterns^41^ than other domestic animals^42,43^.

Furthermore, studies directly comparing referential communicative capacities between species are scarce and suggest that performance differences may be affected by task settings. In unsolvable task settings, only dogs but not similarly raised wolves (*Canis lupus*)^44^*,* companion cats (*Felis catus*)^45^ or companion pigs^46^ exhibited referential communicative behaviours when an easy-to-open box containing a reward became impossible to open. Nevertheless, recent works suggested that performance in the unsolvable task might reflect independence in problem-solving and manipulative biases (i.e. a tendency to physically manipulate objects of interest during exploration) more than referential communicative capacities^47,48^. Indeed, in a direct comparison with family dogs in an out-of-reach reward setting (where independent manipulative attempts to access the reward are never reinforced, and the reward is only accessible to the human present)^49^, wolves also exhibited human-oriented referential communicative behaviours^22^.

Here we use an out-of-reach paradigm to compare human-oriented communicative behaviours in companion dogs and companion pigs. We separately manipulate the presence/absence of the human and the reward: we assume that behaviours that increase in the presence of the human are potentially communicative^50^ and behaviours that increase when both the human and the reward are present are potentially referentially communicative^11^. Finding that pigs exhibit referential communicative behaviours as dogs do would indicate that efficient intraspecific visual communication of a species is not a prerequisite for such behaviours to emerge. However, finding that pigs fail to exhibit such behaviours would suggest that species–specific characteristics may hinder human-oriented referential communication, even in domestic, intensely socialized mammals.

## Materials and methods

### Ethics statement

We obtained written official approval (#PE/EA/430-6/2018) for the experimental protocols from local ethical committees: Állatkísérleti Tudományos Etikai Tanács (Scientific Ethic Council of Animal Experiments); Pest Megyei Kormányhivatal Élelmiszerlánc-biztonsági és Állategészségügyi Igazgatósága, Budapest, Hungary (Food Chain Safety and Animal Health Directorate Government Office). According to this statement and the corresponding definition by law in Hungary, the current non-invasive observational studies are not considered to be animal experiments. We received the necessary permission from the University Institutional Animal Care and Use Committee (UIACUC, Eötvös Loránd University, Hungary). All methods were carried out in accordance with the local committees’ guidelines and reported in accordance with ARRIVE guidelines.

### Subjects

Eleven pigs (7 males, 4 females, age ± SD = 9.63 ± 1.24 months, Minnesota and mixed miniature variants) and thirteen dogs (8 males, 5 females, age ± SD = 8.30 ± 1.49 months, 8 different breeds) participated. The original sample size for pigs was also thirteen, but two animals had to be excluded due to owners’ constraints. Former research has shown that socialized individuals from both species perform human-oriented communicative behaviours already at 4 months of age^40^, therefore investigating the spontaneous emergence of referential communication towards humans in these animals at 8 months of age seemed appropriate. All subjects were living in human families from ~ 8 weeks old and experienced similar socialization. For subject and recruitment details, see Supplementary Information and Supplementary Table S1. All owners signed a written informed consent form to participate in the study, publish their images and data in open access, and voluntarily permit their pigs and dogs to participate in the study.

### Procedure

**Figure 1:**
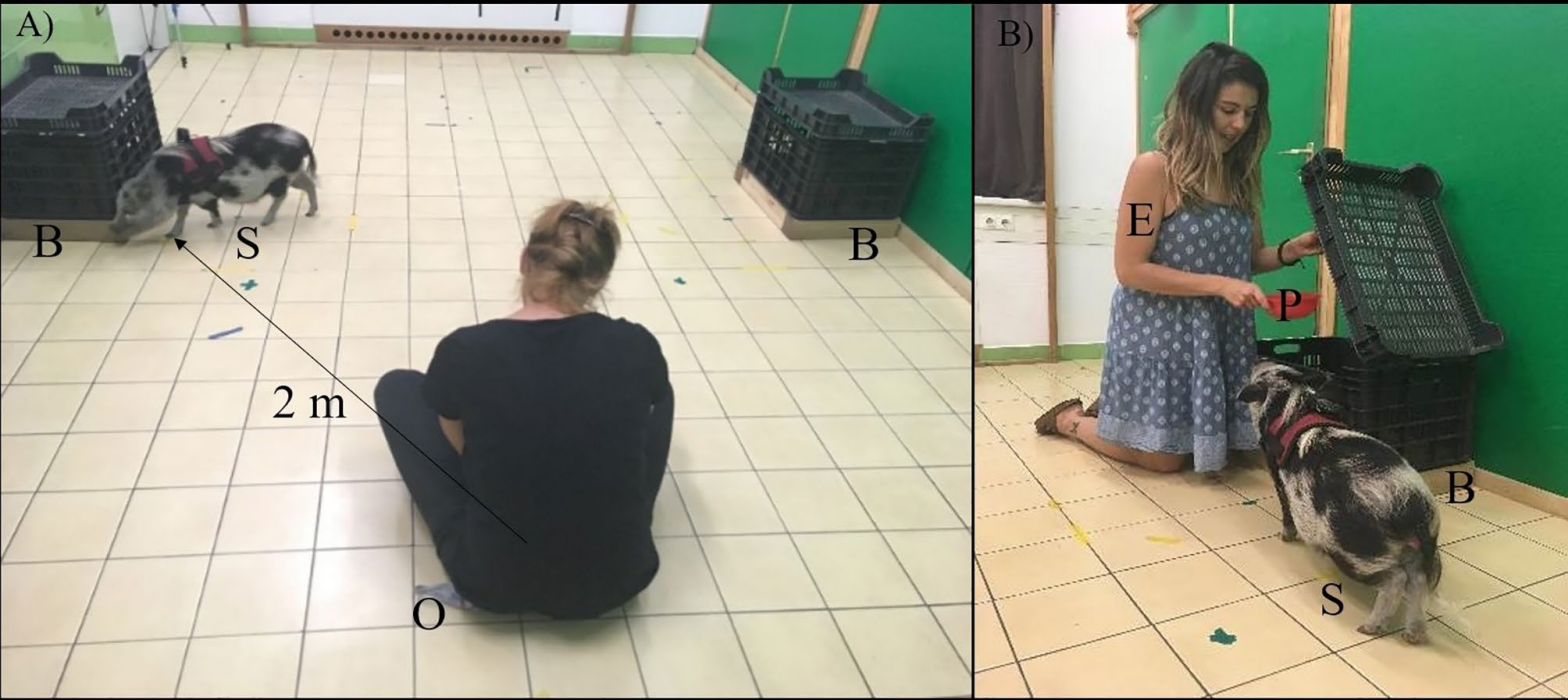
Experimental setup. *S* Subject, *O* owner, *B* Hiding boxes, *E* experimenter, *P* plastic container. (**A**) Setup of the test, (**B**) Experimenter hiding the food reward.

The experiment was performed in a room (4.45 × 3.68 m) of the Department of Ethology (Eötvös Loránd University, Budapest). Two identical black, holed, plastic boxes (43 × 37 × 59 cm) which could be opened only from the upper part were placed on the two sides of the room (Fig. 1). Inside each of the boxes, there was a red plastic container, visible but impossible to reach for the subject. Each box was a possible food hiding location.

The study consisted of a *Training phase* followed consecutively by an *Experimental phase* (based on Miklósi et al.^49^). In the *Training phase,* we showed the subject (S) the possible food locations and that only a human could open the boxes. The S, the owner (O), and the experimenter (E) entered the test room, and the S was allowed to walk around off-leash and explore the room. Then the E showed some pieces of food to the S: sausages for the dogs and dog food for the pigs—we chose that food type that proved to be of similarly high value for the individuals based on owner reports. The E placed the food in one of the red plastic containers, which was first on the floor, and let the S eat from it. Then the E dropped again a few pieces of food in the red plastic container, and placed it inside one of the boxes, letting the S smell from outside and making sure that the S was noticing the food inside the box, although only the container and not the food was visible from outside. After this, the E and the O opened the box together and gave the food to the S. The training was repeated the same way with the other red plastic container and with the other black plastic box. The order of sides was randomized. Then the red containers were placed inside each box and the S, the O, and the E left the room.

The *Experimental phase* consisted of 3 conditions the order of which was balanced across subjects: (a) *Owner Condition:* provided a baseline measure for human-oriented behaviours in the absence of any desired target; (b) *Food Condition:* provided a baseline measure for food-oriented behaviour; and (c) *Food* + *Owner Condition*: allowed the investigation of referential communicative behaviours in the presence of both the owner and the food.

Each condition was divided into *Habituation, Treatment,* and *Test,* and all started with the E opening the corridor door for the S and the O to enter the room. Then the O sat in a fixed position at a 220 cm distance from both boxes with his/her back to the door (Fig. 1). The O remained passive during the whole procedure to avoid any unintentional influence on the emergence of the subjects’ signalling behaviour.

- *Habituation:* Same for all 3 conditions. The O sat at her/his position, read a mobile/journal, and remained passive. The S was free to explore for 1 min after which the E knocked on the door and the O left.
- *Treatment*
  - *Owner Condition*: The E entered and petted the S for 30 s and then left.
  - *Food Condition* and *Food* + *Owner Condition*: The E entered with food and showed it to the S. The E approached one of the boxes, picked up the red container and placed the food inside, making sure that the S followed the location. Then the E placed the container inside the designated box. The position of the box containing the food (left of right) was counterbalanced across subjects and conditions. Finally, the E left the room.
- *Test*
  - *Owner Condition* and *Food* + *Owner Condition:* The O entered, sat at the designated place, ignoring the S, and reading. After 1 min, the E entered the room.
  - *Food Condition*: The S was alone in the room for 1 min after which the O and the E entered the room.

### Behavioural analysis

All tests were video-recorded and analysed using Solomon Coder (v. 19.08.02; András Péter, http://solomoncoder.com). The observation started when the O sat down in the *Test* of all Conditions except for the “*Food Condition*”, in which it started after the E closed the door (60 s in all cases). We measured the duration of vocalizations; duration of orientation and interaction towards/with the boxes (in “*Food Condition*” and “*Food* + *Owner Condition*” it is the sum of the S’s orientation or interaction towards/with the box containing food and the empty box) and the proportion of time interacting with the boxes meanwhile orienting towards them. When the owner was present (“*Owner Condition*” and “*Food* + *Owner Condition*”*)*, we measured the duration of orientation and interaction towards/with the owner, and the frequency of alternation of orientation between the owner and the boxes (S orients its head towards any of the boxes followed or preceded by orienting towards O within a maximum of 2 s). When the owner was absent (“*Food Condition*”) we measured the duration of orientation towards the door and the frequency of alternation of orientation between the door and the boxes (see Supplementary Table S2 for the behavioural variables’ definitions). One person coded all videos, and a secondary coder, blind to the study’s objective, double-coded 20% of them. We used the raw coding sheets to calculate inter-rater agreement for “Orientation” (towards boxes, human, and door) and “Interaction” (with boxes and human) where the occurrence of these behaviours was marked every 0.2 s. Analysis indicated acceptable reliability (mean ± SD Cohen’s kappa: orientation 0.85 ± 0.06; interaction 0.86 ± 0.02). Agreement for “Alternation of orientation" proved to be strong (Spearman’s rank correlation, r_s_ = 0.88, p < 0.001).

### Data analyses

We used the R statistical environment (v. 4.2.1^51^) to carry out statistical analyses (details in Supplementary R script). Condition effects were investigated by comparing *Test* phases of the conditions (“*Food*” vs. “*Food* + *Owner”*, “*Owner*” vs. “*Food* + *Owner*”). We applied Generalized Linear Mixed Models using Template Model Builder (GLMMs using TMB, R package “glmmTMB”^52^) to analyse orientation towards and interaction with the boxes. In the majority of conditions less than half of the dogs vocalized, whereas more than 2/3 of the pigs vocalized in every condition, therefore duration of vocalization was analysed only in pigs. Occurrence of vocalization (binomial variable) was calculated for both species, and it was analysed in binomial Generalized Linear Mixed Models (binomial GLMMs, R package “lme4”^53^). Models included condition and species as fixed factors and animals’ ID as random term. Likelihood ratio tests (LRT) were conducted to investigate the significance of the explanatory variables in the models. We report χ^2^ and the corresponding p-values of LRTs and parameter estimates (β ± SE) for significant explanatory variables. We also provide contrast estimates (β ± SE) from post hoc analyses (R package “emmeans”^54^; Tukey correction), which were performed for multiple comparisons. The rest of the behaviours were analysed in non-parametric tests due to the distribution of the variables. Interaction during orientation towards the boxes, orientation towards the door/owner, alternation between the boxes and the door/owner, and duration of pig vocalization were analysed in Mann–Whitney U Tests (species effect) and Friedman Tests (condition effect). Interaction with the owner was analysed in Mann–Whitney U Tests (species effect) and Wilcoxon Signed Rank Tests (condition effect). Durbin-Conover post hoc tests were performed for pairwise comparisons, and we controlled for multiple comparisons by adjusting p-values using Holm’s method.

## Results

**Figure 2:**
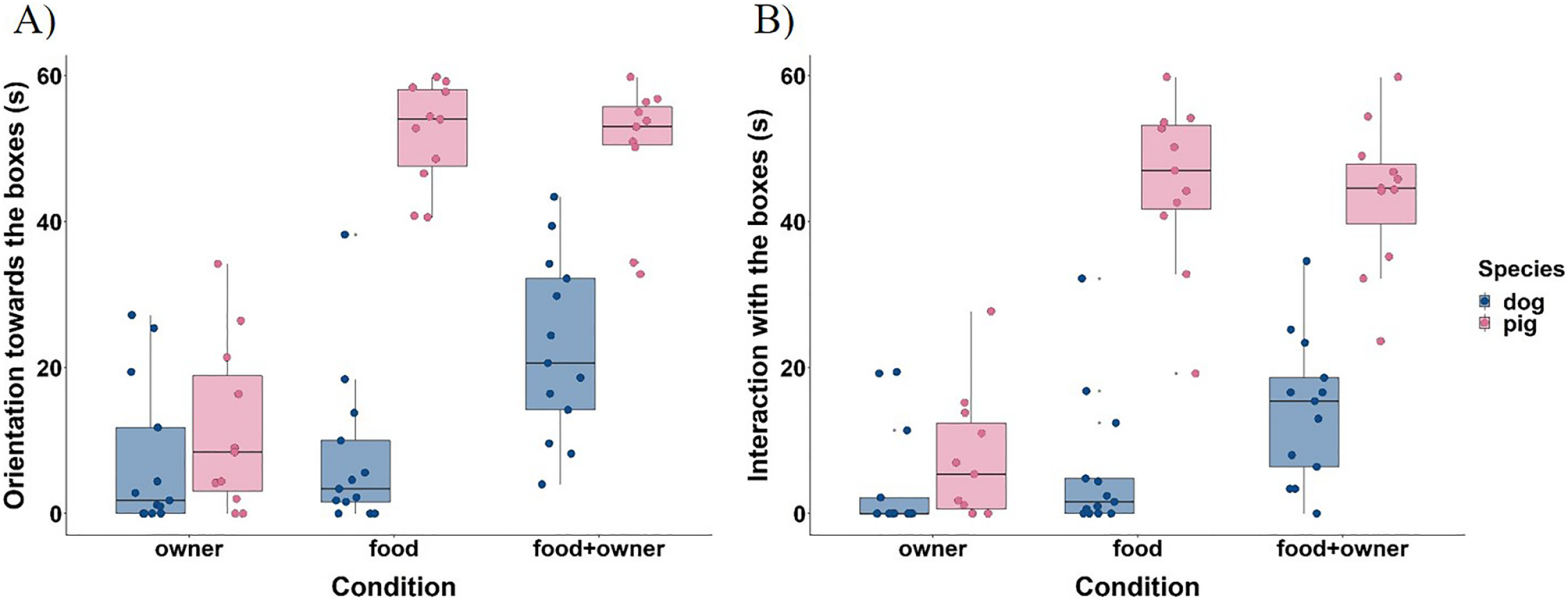
Time spent orienting towards the boxes (**A**) and interacting with them (**B**) for both species. Boxplots indicate the median, 25th and 75th percentiles (boxes), and the minimum and maximum (whiskers). Dots represent individual data points.

Table 1 summarizes all significant effects (for detailed statistical test information see Supplementary Table S3). Dogs exhibited more orientation and interaction towards/with the boxes in the “*Food* + *Owner Condition*” than if only the food or only the owner was present. If the food was present (“*Food Condition*” *and* “*Food* + *Owner Condition*”*)*, pigs exhibited these behaviours to a similar extent and more often when both the food and the owner were present (“*Food* + *Owner Condition*”) than if the food was absent (“*Owner Condition*”*).* Pigs also oriented towards and interacted with the boxes more often than dogs whenever the food was present, irrespective of the human’s presence. Besides, pigs interacted similarly with the boxes while orienting towards them in the “*Food Condition*” compared to the “*Food* + *Owner Condition*”*,* but they performed this behaviour more in the “*Food* + *Owner Condition*” than in the “*Owner Condition*”*.* Moreover, we found a trend that pigs displayed this behaviour more than dogs if only the food was present (“*Food Condition*”*)* (Fig. 2).

**Table 1:** Condition and species effects. Results of Durbin-Conover post hoc tests, Mann–Whitney U Tests and pairwise comparisons of Generalized Linear Mixed Models.

Condition effects Dogs			
**Food vs. food + owner**			
<	Orientation towards the boxes	p < 0.001	β ± SE = − 1.45 ± 0.34, t = − 4.30
<	Interaction with the boxes	p = 0.008	β ± SE = − 1.02 ± 0.33, t = − 3.12
>	Orientation towards the door/owner	p < 0.001	df = 12
<	Alternation between the boxes and the door/owner	p < 0.001	df = 12
**Owner vs. food + owner**			
<	Orientation towards the boxes	p < 0.001	β ± SE = − 1.60 ± 0.35, t = − 4.63
<	Interaction with the boxes	p < 0.001	β ± SE = − 1.49 ± 0.36, t = − 4.15
<	Alternation between the boxes and the owner	p < 0.001	df = 12
**Pigs**			
**Food vs. food + owner**			
<	Orientation towards the door/owner	p = 0.003	df = 10
<	Alternation between the boxes and the door/owner	p = 0.022	df = 10
**Owner vs. food + owner**			
<	Orientation towards the boxes	p < 0.001	β ± SE = − 3.13 ± 0.39, t = − 8.05
<	Interaction with the boxes	p < 0.001	β ± SE = − 3.05 ± 0.35, t = − 8.69
<	Interaction during orientation towards the boxes	p < 0.001	df = 8
>	Orientation towards the owner	p = 0.006	df = 10

Species effects Dogs vs. pigs			
**Food condition**			
<	Orientation towards the boxes	p < 0.001	β ± SE = − 3.70 ± 0.44, t = − 8.45
<	Interaction with the boxes	p < 0.001	β ± SE = − 3.43 ± 0.42, t = − 8.12
<	Interaction during orientation towards the boxes	p = 0.053	U = 23, n_d_ = 10, n_p_ = 11
>	Orientation towards the door	p < 0.001	U = 143, n_d_ = 13, n_p_ = 11
**Owner condition**			
<	Occurrence of vocalization	p = 0.017	β ± SE = − 4.62 ± 1.94, z = − 2.38
**Food + owner condition**			
<	Orientation towards the boxes	p < 0.001	β ± SE = − 2.08 ± 0.39, t = − 5.29
<	Interaction with the boxes	p < 0.001	β ± SE = − 2.30 ± 0.40, t = − 5.80
>	Orientation towards the owner	p = 0.002	U = 132, n_d_ = 13, n_p_ = 11
>	Alternation between the boxes and the owner	p = 0.002	U = 129, n_d_ = 13, n_p_ = 11

**Figure 3:**
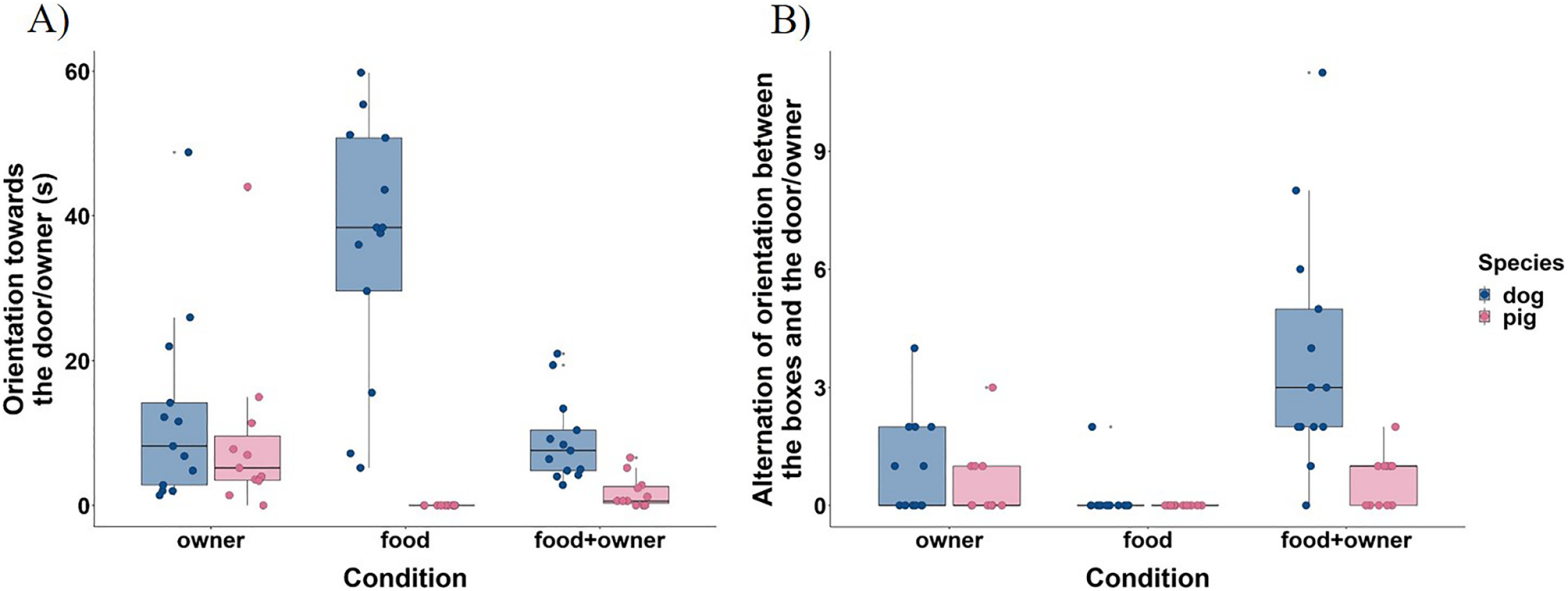
Time spent orienting towards the door/owner (**A**) and frequency of alternation of orientation (**B**) for both species. Boxplots indicate the median, 25th and 75th percentiles (boxes), and the minimum and maximum (whiskers). Dots represent individual data points.

Dogs exhibited more orientation towards their owner/door in the “*Food Condition*” than in the “*Food* + *Owner Condition*”. If the owner was present (“*Owner Condition*” *and* “*Food* + *Owner Condition*”) dogs oriented to him/her for a similar amount of time*.* Pigs' owner orientation changed gradually across conditions; it was the most frequent in the absence of food and least frequent in the absence of the owner (in the “*Food condition*” we used as owner orientation the orientation towards the door through which the owner had left). If food was present (“*Food Condition*” *and* “*Food* + *Owner Condition*”*)* dogs oriented more towards their owner than pigs (Fig. 3A). We found no condition or species effects for the time animals spent interacting with their owner.

Dogs exhibited more orientation alternation between the boxes and the owner in the “*Food* + *Owner Condition*” than if only the food or only the owner was present. Compared to the “*Food* + *Owner Condition*” pigs alternated similarly when only the owner was present (“*Owner Condition*”*),* and less in the “*Food Condition*”. Besides, pigs alternated less than dogs in the “*Food* + *Owner Condition*” (Fig. 3B).

Condition had no effect either on the number of vocalizing dogs and pigs or on the overall duration of pig vocalizations. However, significantly more pigs than dogs vocalized in the “*Owner Condition*”.

We calculated effect sizes for all our comparisons (R package “effectsize”^55^). All of the calculated Cohen’s ds > 0.70.

## Discussion

Comparing the behaviour patterns of young family pigs and dogs in an out-of-reach paradigm, we found evidence for human-oriented referential communication in dogs only. In the concurrent presence of the human and the target, dogs’ alternations of orientation and their target-oriented behaviours selectively increased, replicating previous work on adult dogs^49^. Conversely, we found no similar behavioural pattern indicative of referential communicative function in pigs. The lack of referential communicative behaviours in pigs is not caused by a general absence of human-oriented communicative behaviours. Indeed, the amount of time pigs spent owner-oriented and also the number of orientation alternations increased in the human’s presence, in line with previous work^46^. Moreover, pigs and dogs showed a similar amount of human-oriented behaviours when no reward was present, confirming a comparable readiness of the two species to spontaneously attend to humans^40^.

Other species differences in behaviour reflected general predispositions^25,40^ rather than specific human-oriented communicative abilities. In the absence of the owner, dogs exhibited more door orientation than pigs, perhaps reflecting separation distress^56^ and their greater dependency on humans^57^. Contrarily, in general, more pigs than dogs vocalized, and pigs oriented towards the reward more than dogs, revealing their highly vocal nature and generally stronger food motivation^58,59^. Nevertheless, no human-oriented communicative intention was apparent in pigs in the patterning of either of these behaviours.

The lack of human-oriented referential communication in pigs may be related to their scarce use of visual social signals for within-species communication that, in turn, may be caused by anatomical constraints including poor vision and neck rigidity^37,38^. Notably, however, other behaviours (e.g. vocalizations), unaffected by pigs' visual capacities, also did not increase in the simultaneous presence of the human and the reward. Another, not mutually exclusive explanation is that even though the out-of-reach paradigm we used here, unlike the unsolvable task^44^, does not reinforce physical interaction with the target reward, pigs’ strong manipulative bias (as reflected in the time spent interacting with the boxes) may have suppressed their willingness to referentially communicate with the human. Future research on this topic including pigs should attempt to fully exclude chances for physical manipulation by for example including elevated out-of-reach rewards.

One might argue that the inattentiveness of the owner could have limited the communicative performance of the subjects. Indeed, visual contact with and visual reassurance from the human partner are known to increase animals’ human-oriented communication^19,60^. In the current study, we decided on an inattentive owner to prevent his/her behaviour from influencing the animals’ spontaneous communicative pattern. As we hypothesized that dogs are especially sensitive to visual communication, having a visually attentive owner could have more strongly affected the behaviour of dogs than of pigs. This could have confounded our results and made it challenging to discern to what extent the emergence of referential communication was spontaneous or affected by the owners' visual response. Furthermore, the inattentiveness of the owner did not make human-oriented communicative behaviours impossible: we found similar behavioural patterns in dogs as in previous research^11^.

The current study presents potential limitations that could restrict the interpretation of our findings. First, the sample size, even though comparable to recent studies on miniature pigs^61,62^, is relatively small, warranting caution in generalizing the results. Second, one potential alternative explanation for the found absence of human-oriented referential communication in pigs could be that this ability emerges later in development in this species. However, companion pigs have been reported to spontaneously show clear human-oriented communicative signs (not referential though) at already 4 months of age^40^, thus this account seems improbable. Furthermore, we cannot exclude that the different selection pressures acting during the two species' domestication (i.e. dogs' selection for close cooperation with humans^63^ vs. pigs’ selection as livestock^28^) have influenced the current findings. To better disentangle the independent and interacting effects of domestication and species-specific characteristics, further studies might include domestic and non-domestic animals which do and which do not rely on visual social cues for intraspecific communication.

To conclude, we demonstrated that an out-of-reach context, which elicits human-oriented referential communicative behaviours in family dogs (and wolves^22^), does not elicit such behaviours in family pigs. We propose that pigs’ failure to referentially communicate with humans indicates their lack of readiness to do so. This, in line with previous results, suggests that domestication or intense human socialization are not sufficient for human-oriented referential communication to emerge in social mammals and that other pre-existing species characteristics such as reliance on visual social signals in intraspecific interactions may have a more determinant role.

## Supplementary Information

Supplementary Information 1.

Supplementary Information 2.

## Data Availability

All data generated and analysed during this study are included in this published article and in its Supplementary Information files (see Supplementary Information, Supplementary Data and Supplementary R script).
