# Supplementary material for: Out-of-reach rewards elicit human-oriented referential communicative behaviours in family dogs but not in family pigs
Source: Sci Rep. 2023 Jan 23;13:811. doi: 10.1038/s41598-022-26503-5 (PMC9871027; doi:10.1038/s41598-022-26503-5)
Supplement: Supplementary file 1 — Supplementary Information 1. [file 41598_2022_26503_MOESM1_ESM.pdf]

Supplementary Information for the manuscript entitled

Out-of-reach rewards elicit human-oriented referential communicative behaviours in family dogs but not in family pigs.

Authors and affiliations

Paula Pérez Fraga[12*], Boglárka Morvai[1 2], Linda Gerencsér[1 2], Fanni Lehoczki[1 2], Attila Andics[12]

1 Department of Ethology, Eötvös Loránd University (ELTE), Hungary

2 MTA-ELTE ‘Lendület’ Neuroethology of Communication Research Group, Hungarian Academy of Sciences – Eötvös Loránd University, Budapest, Hungary

* Correspondence: pauliperezfraga@gmail.com

Detailed subject information

All the subjects of this study were living in human families and exposed to intensive human contact from the age of - 8weeks. The pigs are part of a long-term scientific project (http://etologia.elte.hu/en/lendulet-neuroethology-of-communication/), which required a strict selection procedure of the families who volunteered to cooperate with the Department of Ethology, Eötvös Loránd University in the long term. They are the same subjects as in previously published works (1,2) so an extended description of the rearing condition and owner selection process can be found there. The dogs’ owners were regular volunteers of the Family Dog Project (https://familydogproject.elte.hu/). Before enrolment, we asked the owners about the socialization background of the dogs to ensure that it was similar to that of pigs. Owners provided a written consent form to permit their dogs and pigs to participate in the study, as well as to publish their data and images.

Subject ID	Species	Birth date	Test age (months)	Gender	Breed
1	Pig	27/10/2017	8	female	Minnesota*
2	Pig	24/10/2017	8	female	Minnesota
3	Pig	14/08/2017	10	male	Minnesota Mixed*
4	Pig	23/10/2017	8	male	Minnesota
5	Pig	19/08/2017	10	male	Minnesota
6	Pig	15/05/2017	12	female	Minnesota Mixed

7	Pig	05/04/2018	10	male	Minnesota Mixed
8	Pig	23/08/2017	10	male	Minnesota
9	Pig	25/04/2018	10	female	Minnesota
10	Pig	29/09/2017	9	female	Minnesota
11	Pig	25/03/2018	11	male	Minnesota
12	Dog	20/06/2018	9	male	Alaskan* Malamute
13	Dog	11/01/2018	7	female	Schnauzer
14	Dog	06/01/2018	9	male	Dachshund
15	Dog	01/01/2017	7	female	Mixed
16	Dog	05/11/2017	8	female	Beagle*
17	Dog	04/10/2017	8	male	Beauceron
18	Dog	15/02/2018	7	male	Dachshund
19	Dog	27/04/2018	10	male	Whippet
20	Dog	27/06/2018	9	male	Pumi*
21	Dog	15/04/2017	12	male	Labradoodle
22	Dog	02/04/2018	7	female	Mixed
23	Dog	12/11/2017	7	male	Boston terrier
24	Dog	28/03/2018	8	Female	Border collie

Table S1. Subject information

*Subjects used for inter-observer agreement.

Behavioural variables

We measured the following behaviours from the moment the owner sat down in the *Test* of all Conditions except from the *Test* in the *Food Condition* in which we measured it after the E closed the door (60 s in all cases).

**Behavioural Variables**	**Definition**
Vocalization (duration, s):	S is emitting any vocalizations (e.g., barks, whines for dogs, grunts for pigs). Measured in *Test* of each condition.
Occurrence of vocalization (binomial variable):	Number of individuals emitting any vocalizations (e.g., barks, whines for dogs, grunts for pigs). Measured in *Test* of each condition.

Orientation to the boxes (duration, s):	S orients its head to any of the boxes, from a static position or meanwhile moving, with or without establishing physical contact with any of the boxes. Measured in *Test* of *"Owner Condition".* In *Test* of *"Food Condition"* and *"Food + Owner Condition"* it is the sum of S's orientation to the correct box (the one containing the food) and the empty box, although orientation to the correct box highly correlates with orientation to any of the boxes (Food Cond.: $r_s = 1$, $N = 24$, $p < 0.001$; Food+Owner Cond.: $r_s = 1$, $N = 24$, $p < 0.001$).
Orientation to the owner (duration, s):	S orients its head from a static position or meanwhile moving, with or without establishing physical contact with the owner. Measured in *Test* of *"Owner Condition"* and *"Food+ Owner Condition"*.
Orientation to the door (duration, s)	S orients its head to the door, from a static position or meanwhile moving, with or without establishing physical contact with it. Measured in *Test* of *"Food Condition".*
Interaction with the boxes (duration, s):	S establishes physical contact with any of the boxes. Measured in *Test* of *"Owner Condition"*. In *Test* of *"Food Condition"* and *"Food + Owner Condition"* it is the sum of S's interaction with the correct box (the one containing the food) and the empty box, although interaction with the correct box highly correlates with interaction with any of the boxes (Food Cond.: $r_s = 1$, $N = 24$, $p < 0.001$; Food+Owner Cond.: $r_s = 1$, $N = 24$, $p < 0.001$).
Interaction ratio with the boxes (value between 0 and 1):	The ratio of interaction with the boxes out of the total time spent orienting towards the boxes. Measured in *Test* of all conditions.
Orientation-alternation (frequency):	Orienting to any of the boxes in *"Owner condition",* and orienting to the correct box in *Test* of *"Food + Owner condition"* (S orients its head towards the box from a static position or meanwhile moving, with or without physical interaction) followed or preceded within a maximum of 3 s by orientating towards O.
Orientation-alternation with the door (frequency):	Orienting to the correct box in *Test* of *"Food condition"* (S orients its head towards the correct box from a static position or meanwhile moving, with or without physical interaction) followed or preceded within a maximum of 2 s by orienting towards the door.

Table S2. Behavioural variables we measured during *Test* of the different conditions.

Statistical analyses

Orientation towards and interaction with the boxes were analysed in Generalized Linear Mixed Models using Template Model Builder. Interaction during orientation towards the boxes, orientation towards the door/owner, alternation between the boxes and the door/owner, and duration of pig vocalization were analysed in Mann-Whitney U Tests (species effect) and Friedman Tests (condition effect). Interaction with the owner was analysed in Mann-Whitney U Tests (species effect) and Wilcoxon Signed Rank Tests (condition effect). Occurrence of vocalization was analysed in binomial Generalized Linear Mixed Models.

Behaviour	Condition effect		Species effect		
	Dogs	Pigs	Food Condition	Owner Condition	Food + Owner Condition
orientation towards the boxes	GLMM using TMB<br>LRT: $\chi^2_2 = 90.49$, $p < 0.001$				
interaction with the boxes	GLMM using TMB<br>LRT: $\chi^2_2 = 67.68$, $p < 0.001$				
interaction during orientation towards the boxes	Friedman Test<br>df = 2<br>p = 0.846	Friedman Test<br>df = 2<br>p = 0.002	Mann-Whitney U Test<br>$U = 23$, $n_d = 10$, $n_p = 11$<br>p = 0.053	Mann-Whitney U Test<br>$U = 26.5$, $n_d = 9$, $n_p = 9$<br>p = 0.272	Mann-Whitney U Test<br>$U = 43$, $n_d = 13$, $n_p = 11$<br>p = 0.168
orientation towards the door/owner	Friedman Test<br>df = 2<br>p = 0.002	Friedman Test<br>df = 2<br>p = 0.002	Mann-Whitney U Test<br>$U = 143$, $n_d = 13$, $n_p = 11$<br>p < 0.001	Mann-Whitney U Test<br>$U = 84.5$, $n_d = 13$, $n_p = 11$<br>p = 0.513	Mann-Whitney U Test<br>$U = 132$, $n_d = 13$, $n_p = 11$<br>p = 0.002
interaction with the owner	Wilcoxon Signed Rank Test<br>df = 12<br>p = 0.168	Wilcoxon Signed Rank Test<br>df = 10<br>p = 0.266	-	Mann-Whitney U Test<br>$U = 96.5$, $n_d = 13$, $n_p = 11$<br>p = 0.196	Mann-Whitney U Test<br>$U = 104$, $n_d = 13$, $n_p = 11$<br>p = 0.090
alternation between the boxes and the door/owner	Friedman Test<br>df = 2<br>p < 0.001	Friedman Test<br>df = 2<br>p = 0.053	Mann-Whitney U Test<br>$U = 77$, $n_d = 13$, $n_p = 11$<br>p = 0.463	Mann-Whitney U Test<br>$U = 82.5$, $n_d = 13$, $n_p = 11$<br>p = 0.516	Mann-Whitney U Test<br>$U = 129$, $n_d = 13$, $n_p = 11$<br>p = 0.002
occurrence of vocalization	binomial GLMM<br>LRT: $\chi^2_4 = 15.87$, $p = 0.017$				
duration of vocalization	-	Friedman Test<br>df = 2<br>p = 0.190	-	-	-

Table S3. Statistical analyses results

Alternation setting

The effect of the alternation coding setting was analysed in Friedman Tests. Durbin-Conover post hoc tests were performed for pairwise comparisons, and we controlled for multiple comparisons by adjusting p-values using Holm's method.

The alternation setting contained the maximum length of the gap between two behaviours (in seconds, the first number in the variable levels) and the maximum length of the two consecutive behaviours (in seconds, the second number in the variable levels). We investigated six alternation settings (11, 12, 22, 23, 32, 33). Alternation setting had no effect on the pigs' orientation alternation between the boxes and the owner in any of the conditions (all $p > 0.053$). However, alternation setting affected the orientation alternation of the dogs in all conditions (all $p < 0.001$). In the *Food Condition*, settings in which the maximum length of the gap between two behaviours was 3 sec (32, 33) differed from the other settings (11, 12, 22, 23) (all $p < 0.001$). In the *Owner Condition*, settings in which the maximum length of the gap was 3 sec (32, 33) differed from settings in which the gap was 1 sec (11, 12) (all $p < 0.016$). In the *Food+Owner Condition*, settings in which the maximum length of the gap was 1 sec (11, 12) differed from the settings in which the gap length was higher (22, 23, 32, 33).

Due to that pigs' alternation did not differ under the investigated settings, we chose the setting which we found the most relevant based on previous studies on the topic (3,4) and more indicative of the dynamic aspect of a referential alternation.

Average length of orientation towards target by each species

Orientation towards the owner/door

Dogs		
**condition**	**mean**	**SD**
food	12.65	15.23
owner	3.05	3.16
food+owner	1.83	1.13
Pigs		
**condition**	**mean**	**SD**
food	0	0
owner	7.34	12.37
food+owner	1.8	2.24

Table S4. Average length of orientation towards the owner/door of both species

Orientation towards the bowl/boxes

Dogs		
**condition**	**mean**	**SD**
food	2.57	3.24
owner	2.44	2.45
food+owner	5.22	2.85
Pigs		
**condition**	**mean**	**SD**
food	18.79	14.96

owner	4.23	2.89
food+owner	19.13	14.77

Table S5. Average length of orientation towards the food of both species

References

1. Gerencsér L, Pérez Fraga P, Lovas M, Újváry D, Andics A. (2019) Comparing interspecific socio-communicative skills of socialized juvenile dogs and miniature pigs. Anim Cogn. Jun 29:1–13. https://doi.org/10.1007/s10071-019-01284-z

2. Pérez Fraga P, Gerencsér L, Andics A. (2020) Human proximity seeking in family pigs and dogs. Sci Rep 10(1):1–11. https://doi.org/10.1038/s41598-020-77643-5

3. Miklósi, A., Polgárdi, R., Topál, J. & Csányi, V. Intentional behaviour in dog-human communication: An experimental analysis of 'showing' behaviour in the dog. *Anim. Cogn.* **3**, 159–166 (2000). https://doi.org/10.1007/s100710000072.

4. Nawroth, C., Brett, J. M. & McElligott, A. G. Goats display audience-dependent human-directed gazing behaviour in a problem-solving task. *Biol. Lett.* **12**, (2016). https ://doi.org/10.1098/rsbl.2016.0283
